# The Interplay Between DNA Repair and the Immune Microenvironment in Pancreatic Cancer

**DOI:** 10.3390/biomedicines13051031

**Published:** 2025-04-24

**Authors:** Aaron Ciner, Peter J. Hosein, Yixing Jiang, Feyruz Rassool

**Affiliations:** 1 Greenebaum Comprehensive Cancer Center, School of Medicine Baltimore, University of Maryland, Baltimore, MD 21201, USA; 2 Sylvester Comprehensive Cancer Center, University of Miami, Miami, FL 33136, USA; phosein@med.miami.edu

**Keywords:** pancreatic cancer, BRCA mutation, homologous recombination deficiency, immune resistance, immunotherapy

## Abstract

This narrative review describes the relationship between DNA repair and the immune microenvironment in pancreatic cancer and its potential clinical relevance. Pancreatic cancer is a devastating disease, often diagnosed at an advanced and incurable stage. BRCA or PALB2 mutations occur in a small subset, disabling accurate DNA double-strand break repair and sensitizing tumors to platinum-based chemotherapy and poly-ADP ribose polymerase inhibitors. While immune checkpoint blockade targeting PD1 and CTLA4 is ineffective for most patients, accumulating translational work indicates that those with BRCA or PALB2 mutations harbor a distinct and more permissive immune microenvironment. The phase 2 TAPUR study and retrospective series demonstrate that combined PD1 and CTLA4 inhibition can be effective for this subgroup of patients. In this manuscript, we review the current treatment landscape, the underlying mechanisms for immune resistance, and the interplay between defective DNA repair and the immune microenvironment in pancreatic cancer.

## 1. Current Treatment Landscape in Pancreatic Cancer

### 1.1. Chemotherapeutic Approaches in Pancreatic Cancer

Pancreatic ductal adenocarcinoma (PDAC) is the third leading cause of cancer-related death in the United States. Approximately 60,000 people are diagnosed annually, and the five-year survival is dismal at 13% [1]. Most individuals present with locally advanced or metastatic disease; a setting in which treatment options are limited. Chemotherapy remains the standard of care and can reduce symptom burden and prolong survival. Efficacy however is modest with 1-year survival rates under 50% and challenging side effects. Targeted therapies only benefit a minority of patients with unique biomarkers including BRCA, PALB2, BRAF, or other rarer molecular alterations. Furthermore, immunotherapy drugs that target the PD1 or CTLA4 axis and have demonstrated benefit in multiple cancer subtypes are ineffective for the broad population with PDAC. This immune resistance is rooted in a suppressive microenvironment infiltrated by tumor-associated macrophages and other myeloid subsets as well as cytotoxic T-cell dysfunction and exclusion. The two most active chemotherapy regimens are gemcitabine with nab-paclitaxel (GNP) and 5-fluorouracil, oxaliplatin, and irinotecan (FOLFIRINOX). In a phase 3 randomized controlled trial in patients with stage 4 PDAC, GNP improved median overall survival (OS) compared with gemcitabine alone (8.5 versus 6.7 months, HR 0.72, *p* < 0.001), with 1-year survival rates of 35% and 22%, respectively [2]. In a separate phase 3 study that enrolled a slightly fitter and younger patient population and used the same control arm, FOLFIRINOX demonstrated a median OS of 11.1 months with 48% of patients being alive at the 1-year mark [3]. These practice-changing trials improved clinical outcomes but also highlighted the urgent need for improved therapeutics for this disease.

### 1.2. Targeted Therapy in BRCA1-, BRCA2-, or PALB2-Mutated Pancreatic Cancer

Pathogenic alterations in BRCA1, BRCA2, or PALB2 represent the largest targetable subgroup in PDAC. These proteins, in their normal state, play an integral role in repairing double-strand DNA breaks. They promote the recruitment and assembly of a large multi-protein complex to sites of DNA damage and facilitate a high-fidelity repair process called homologous recombination (HR). BRCA1 interacts with multiple regulators including PALB2 to localize to sites of DNA damage and stimulate nuclease activity and RAD51 binding, integral initial steps of HR [4,5]. The primary role of BRCA2 in contrast is later in the repair process and includes the recruitment and positioning of RAD51 to enable the strand invasion of the sister chromatid [6]. Somatic or germline mutations in BRCA1, BRCA2, or PALB2 occur in approximately 10% of patients with pancreatic cancer. The loss of any of these proteins impairs HR and leads to genomic instability by forcing reliance on error-prone DNA repair mechanisms like non-homologous or microhomology-mediated end-joining [7]. A subset of patients with BRCA1, BRCA2, or PALB2 mutations demonstrate remarkable sensitivity to DNA-damaging agents including platinum chemotherapy because of ineffective DNA repair. They also can respond favorably to drugs that target poly-ADP ribose polymerase (PARP), an enzyme that helps repair single-strand DNA breaks. PARP inhibitors (PARPis) target BRCA1-, BRCA2-, or PALB2-altered cancers by trapping PARP in chromatin complexes and impeding the repair of single-strand breaks, leading to replication fork collapse and catastrophic DNA damage [8].

In a phase 2 trial that treated patients with germline BRCA1, BRCA2, or PALB2 mutant PDAC with platinum-based chemotherapy with or without the PARPi, velaparib, radiographic response rates were above 60% and median OS was greater than 15 months in both arms [9]. These results compare favorably with historical radiographic response rates of 20–30% and an anticipated life expectancy of less than 1 year; enhanced outcomes to platinum-based chemotherapy for those with germline BRCA1, BRCA2, or PALB2 mutations are also supported by multiple retrospective analyses [2,3,10,11]. PARPis were the first targeted therapy to improve outcomes in PDAC. In the phase 3 placebo-controlled POLO trial, maintenance olaparib doubled progression-free survival over placebo among patients with a germline BRCA1 or BRCA2 mutation who did not progress on frontline platinum-based chemotherapy. While this did not translate into an improvement in OS, the approximate 25-month duration of response with olaparib in the subgroup of responders clearly pointed to a cohort with significant benefit from this drug class [12]. A smaller phase 2 study with a similar design showed promising results as well. In this trial, maintenance therapy with the PARPi, rucaparib, led to a radiographic response rate of 42%, a median progression-free survival of 13.1 months, and a median OS of 23.5 months [13]. The results of prospective studies using platinum-based chemotherapy and/or PARPis in those with BRCA1-, BRCA2-, or PALB2-mutated PDAC are described in Table 1.

Importantly, not all patients with BRCA1, BRCA2, or PALB2 mutant PDAC respond favorably to platinum-based chemotherapy or PARPi. For example, biallelic inactivation of BRCA or PALB2 is a critical indicator of HR deficiency (HRD) and sensitivity to DNA-damaging agents. In one database analysis, those with stage 4 PDAC with biallelic BRCA1 or BRCA2 mutations had a median survival of 26 months after platinum-based chemotherapy compared to 8.6 months for the subgroup with only monoallelic inactivation [14]. A separate study aimed to measure the relationship between biallelic BRCA loss and HRD in a cohort of patients with germline BRCA mutation and a diagnosed cancer. Using the HRDex tool to measure the degree of genomic scarring, 81% of those with biallelic BRCA1 or BRCA2 loss harbored high HRD scores (HRDex > 42) while only 22% exhibited high HRD with monoallelic inactivation [15]. On the other hand, certain patients with pancreatic cancer who do not have BRCA or PALB2 mutations still maintain an HRD phenotype and can benefit from platinum-based chemotherapy or PARPi. For example, in a large retrospective pancreatic cancer analysis, Golan and colleagues detected an HRD or BRCAness phenotype in approximately 10% of those without a germline BRCA or PALB2 variant [16]. Whether composite genomic scar scores like Myriad’s HRD assay, distinct mutational signatures, functional RAD51 foci formation, or another method can outperform the presence of BRCA or PALB2 mutations to predict BRCAness and platinum or PARPi sensitivity remains uncertain. Optimally characterizing the subgroup with HRD that will benefit most from these drug classes is an area of active investigation [17].

## 2. Immunotherapy and the Immune Microenvironment in Pancreatic Cancer

### 2.1. Immune-Based Strategies in Pancreatic Cancer

While multiagent chemotherapy and targeted therapy broadened the treatment landscape for patients with PDAC, immunotherapy has failed to demonstrate meaningful benefit. The first FDA-approved drugs in this class were monoclonal antibodies targeting CTLA4 and PD1. These agents, termed checkpoint inhibitors, facilitate T-cell priming, trafficking to the tumor microenvironment, and cytotoxic anti-cancer activity and lead to durable responses for patients with cutaneous melanoma, non-small-cell lung cancer, and renal cell carcinoma, amongst other malignancies. PD1 and CTLA4 inhibitors, however, show minimal efficacy in unselected patients with PDAC. In a phase 1 study evaluating the PD1 inhibitor pembrolizumab by Brahmer and colleagues, 14 patients with advanced PDAC were treated and none showed a radiographic response [18]. In a separate phase 2 study of single-agent ipilimumab, a monoclonal antibody against CTLA4, in patients with locally advanced or metastatic PDAC, 0 out of 27 patients demonstrated a radiographic response [19]. Another phase 2 trial evaluated the PDL1 antibody durvalumab alone or combined with the CTLA4 inhibitor, tremelimumab, in a similar patient population. Again, no responders were seen in the monotherapy arm while 1 out of 32 patients had a radiographic response in the doublet arm [20]. Combination strategies with immunotherapy added to a chemotherapy backbone have also been disappointing [21,22,23]. Traditional biomarkers of immune responsiveness like a high tumor mutational burden (TMB) and mismatch repair deficiency (dMMR) that are associated with T-cell infiltration and a ‘hot’ tumor microenvironment (TME) are rare in PDAC. Even in the subset with dMMR, outcomes are worse for those with pancreatic cancer compared to other solid tumors. In a phase 2 study using pembrolizumab in dMMR non-colorectal cancers, the radiographic response rate and median OS were 18% and 4 months in PDAC. Other gastrointestinal cohorts including patients with gastric, small intestinal, and biliary tract cancer demonstrated radiographic response rates greater than 40% and a median OS greater than 2 years with pembrolizumab [24]. Novel immunotherapeutic approaches targeting alternative checkpoints, as well as vaccine-based and cellular strategies, are under active investigation [25,26,27,28]. Since PD1 and CTLA4 blockade are ineffective in the broad pancreatic cancer population, it is important to consider the underlying causes of resistance and potential subgroups that might be more sensitive to an immune-based approach.

### 2.2. The Immunosuppressive Microenvironment in Pancreatic Cancer

Immune resistance in PDAC is mediated by tumor cells with low TMB and antigenicity, in addition to a suppressive TME composed of a desmoplastic, hypo-vascular stroma with high interstitial pressures. Amongst cancer subtypes, pancreatic cancer generally maintains a low tumor mutational burden and consequently a low number of neoantigens [29]. The quality of neoantigens is also important in establishing an effective anti-cancer T-cell response [30]. Only 10–20% of a pancreatic tumor mass represents cancer cells while the majority is composed of an extracellular matrix, cancer-associated fibroblasts, and cells of the innate and adaptive immune system that facilitate local growth and metastatic spread [31]. Tumor-associated macrophages (TAMs) play a major role in the suppressive pancreatic TME. While they exhibit significant plasticity and function along a continuum, they are broadly categorized into two opposing states—M1 and M2. M1 macrophages secrete pro-inflammatory cytokines, activate antigen-presenting cells, and trigger anti-cancer T-cell activity. M2 macrophages in contrast promote angiogenesis, the degradation of the extracellular matrix, and immune evasion through the release of immunosuppressive growth factors, cytokines, and proteases. These pro-tumorigenic TAMs predominate in the pancreatic TME and TAM infiltration positively correlates with patient prognosis in multiple retrospective analyses [32,33]. IL10^+^MHCII^low^ TAMs also increase T-helper 2 (T_H_2) cell differentiation and downregulate cytotoxic CD8^+^ T-cell responses [34,35]. Myeloid-derived suppressor cells (MDSCs) and tumor-associated neutrophils (TANs) contribute to immune resistance as well. MDSCs are immature myeloid cells that promote tumorigenesis across several cancer subtypes. In PDAC, Bayne and colleagues highlighted the role of granulocyte–macrophage colony-stimulating factor in MDSC recruitment, T-cell dysfunction, and tumor growth in the KPC mouse model [36]. MDSCs also secrete immunosuppressive cytokines including IL-10 and TGF_β_, and express inhibitory checkpoints like PD1, which contribute to impaired NK- and T-cell-mediated cytotoxicity [34]. TANs can promote T-cell dysfunction through the release of IL-10 and TNF_α_, and facilitate tumor invasion and metastatic spread via growth factors, matrix metalloproteinases, and reactive oxygen and nitrogen species [37]. TANs also produce web-like scaffolds composed of DNA fibers and proteins called neutrophil extracellular traps (NETs). Ordinarily, NETs protect against bacterial and fungal infections, but these structures in the TME increase liver metastases and T-cell exclusion [37,38,39].

Multiple translational studies highlight the role of KRAS and TP53, the most common driver mutations in PDAC, in facilitating the influx of suppressive myeloid cells and excluding cytotoxic T cells from the pancreatic TME [40,41]. T cells comprise multiple subgroups including CD8^+^ cytotoxic T cells, CD4^+^ T_H_2, T_H_1, and T_H_17, and regulatory T cells (T_regs_). These distinct populations interact with each other and the innate immune system to influence tumor progression. Cytotoxic T cells represent the most potent anti-cancer immune cell. To exert tumoricidal activity, they must be present in the TME, engaged by antigen-presenting cells (APCs) through MHC presentation, and activated by costimulatory receptors. Cancers can evade immune surveillance, however, by excluding cytotoxic T cells from the TME, impairing antigen presentation, or facilitating T-cell dysfunction and exhaustion [42]. In PDAC, CD8^+^ T cells often reside in the surrounding stroma, spatially distant from tumor cells [43,44]. More recent analyses using multiplex immunohistochemistry and single-cell RNA sequencing in human PDAC samples point to patient heterogeneity in the degree of tumor T-cell infiltration. Regardless of the presence of effector T cells in proximity to the tumor, these reports highlight T cells’ downregulation of perforin and other cytotoxic elements and the expression of inhibitory receptors like LAG3 and TIGIT that leads to an exhausted phenotype. Further, the degree of T-cell exhaustion positively correlates with disease stage [44,45]. CD4^+^ regulatory T cells (T_regs_) also contribute to progressive immune dysfunction. T_regs_ express inhibitory checkpoints like CTLA4 and TIGIT and secrete immunosuppressive cytokines that inhibit CD8^+^ T-cell activation and function [46]. The percentage of T_regs_ increases from pre-malignant pancreatic lesions to frank adenocarcinoma and is associated with distant metastasis and worse survival [47]. While much remains unknown about the role of T_regs_ in tumor growth and spread, they contribute alongside TAMs, MDSCs, TANs, and cancer-associated fibroblasts in fostering a hostile tumor-permissive immune TME.

## 3. Relationship Between DNA Repair and the Immune Microenvironment in Solid Tumors

### BRCA Mutation, HRD, and the Immune Microenvironment in Solid Tumors

Even as most patients do not respond to checkpoint inhibition, certain subgroups might harbor a more favorable immune milieu and benefit from an immune-based approach. Impaired HR through mutations in BRCA1, BRCA2, PALB2, or potentially other mechanisms, can increase the rate of point mutations and random insertions and deletions, promoting tumor neoantigen formation and presentation, and cytotoxic T-cell infiltration [48]. Additionally, tumors with HRD are genomically unstable and undergo recurrent chromosomal damage that leaks DNA into the cytoplasm and activates cyclic GMP-AMP synthase (cGAS). This enzyme triggers STING-mediated signaling that upregulates inflammatory cytokines including NFkB and Type 1 interferons [49]. Intriguingly, inflammatory signaling through IFN, TNF, and NFkB can trigger HRD, which is indicative of bidirectional crosstalk between DNA repair and immune activation [50]. In a tumor-agnostic cohort study by Zhou and colleagues, those with BRCA1 or BRCA2 alterations had higher median TMB than their wild-type counterparts (24.6 vs. 5.9, *p* < 0.001) and comprised a higher proportion with high-TMB tumors (defined as the top 10% of TMB in each tumor type). Interestingly, patients with BRCA2 mutations, but not BRCA1, who received an immune checkpoint inhibitor showed a statistically significant increase in median OS compared to those with BRCA wild-type tumors, irrespective of TMB level [51]. Real-world database analyses suggest that BRCA1 or BRCA2 pathogenic variants are associated with increased PDL1 expression on tumor and immune cells and that biallelic inactivation correlates with the highest TMB [52,53]. In translational work using high-grade serous ovarian cancer samples, tumors with mutations in BRCA1 or BRCA2 or other DNA damage response genes exhibited higher neoantigen load, CD3^+^ and CD8^+^ tumor-infiltrating lymphocytes, CD8^+^/CD4^+^ ratio, and PDL1 expression compared to those without mutated genes in the HR pathway [48]. In a small prostate cancer analysis, BRCA2 mutant tumors harbored increased intra-tumoral T-cell infiltration compared to the wild type, although the CD8^+^/FOXP3 ratio trended lower in the mutant subgroup, suggestive of an enhanced immunosuppressive TME [54]. In a landmark study by Samstein and colleagues using 4T1 breast cancer and CT26 colon cancer models, BRCA2 knockout led to CD4^+^ and CD8^+^ T-cell infiltration and increased interferon signaling and NK cell activation. While BRCA2-proficient tumors were resistant to anti-PD1 therapy, the knockout of BRCA2 abrogated tumor growth after immunotherapy treatment. BRCA1 deletion in the 4T1 breast cancer model in contrast led to a distinct and relatively immune-suppressive TME, resistant to single-agent or dual-checkpoint blockade [55]. Even as the reports by Zhou and Samstein suggest that BRCA2 deficiency associates more strongly with an immunogenic phenotype [51,55], other data conflict with this assumption. In a study by George and colleagues using gene expression and copy number analysis in a cohort with high-grade serous ovarian cancer, tumors with BRCA1 alterations harbored significantly increased intra-epithelial T-cell infiltration compared to BRCA2-mutated samples [56]. More research is therefore needed to clarify the effect of distinct BRCA variants and cancer subtype on anti-tumoral immunity.

These pre-clinical findings do not necessarily translate to the clinic. In a small phase 2 study using nivolumab and ipilimumab in women with BRCA mutant ovarian cancer, the radiographic response rate was 22% and the median duration of response was greater than 9 months, suggestive of a signal of activity even though it did not meet the prespecified threshold of efficacy [57]. In a subgroup analysis of a phase 3 randomized trial evaluating the addition of atezolizumab to standard therapy in women with advanced ovarian cancer, there was no increased sensitivity to an anti-PDL1 monoclonal antibody in patients with BRCA1 or BRCA2 mutations or a high genomic loss of heterozygosity score. While those with BRCA1 or BRCA2 pathogenic variants had modestly higher TMB than the wild type (3.78 vs. 2.52 mut/Mb), this did not lead to differences in clinical outcomes between the two groups [58]. More broadly, immune-based approaches have not consistently demonstrated clinical benefit in BRCA-associated cancers including breast, ovarian, and prostate cancer [59,60,61].

## 4. The Impact of HRD on the Immune Microenvironment in Pancreatic Cancer

### 4.1. BRCA Mutation, HRD, and the Immune Microenvironment in Pancreatic Cancer

BRCA mutations or alternative markers of HRD might influence the TME in PDAC as well. In one real-world database study, BRCA2 and PALB2 alterations were enriched in patients with PDAC and tissue TMB ≥ 10 mut/Mb compared to their low TMB counterparts (16% vs. 3.3%) [62]. A separate genomic analysis by Seeber et al. reported increased TMB and higher rates of dMMR in BRCA1-, BRCA2-, and PALB2-mutated PDAC; samples with the BRCA1 or BRCA2 pathogenic variants also expressed PDL1 (defined by ≥1% on tumor cells) more frequently [63]. In a single-institution tissue-based analysis, TMB was particularly high in samples with biallelic BRCA1 or BRCA2 variants and secondary resistance mutations (9.7 mut/Mb) compared to BRCA-null tumors without resistance mutations (4.1 mut/Mb) and HR-proficient tumors (2.5 mut/Mb). In a patient-derived xenograft humanized germline BRCA2-deficent model with an acquired secondary resistance mutation and a TMB of 12.2 mut/Mb, an anti-PD1 monoclonal antibody significantly slowed tumor growth and promoted tumor T-cell infiltration [64]. Golesworthy and colleagues analyzed the immune TME of PDAC samples that were HR-proficient versus HRD (defined as the presence of a BRCA1, BRCA2, or PALB2 germline mutation and an HRDetect score of ≥ 0.9). HRD tumors exhibited increased intra-tumoral CD8^+^ T-cell and T_regs_ infiltration compared to HR-proficient tumors. The proportion of samples that expressed PDL1 on tumor and immune cells was also higher in the HRD subset [65]. Terrero et al. reported that patients with BRCA1, BRCA2, or RAD51 mutations who responded to immunotherapy had higher levels of the T-cell trafficking chemokines CCL4, CCL5, CXCL9, and CXCL10 compared to non-responders [66]. This finding was recapitulated in a mouse model of BRCA2-deficient PDAC, along with a parallel increase in cGAS-STING and Type 1 interferon signaling [67]. Taken together, these results suggest that the HRD defect leads to transcriptional reprogramming of the tumor cells, which shifts the TME balance from an immunosuppressive to an immune-permissive state. In separate experiments by Shaashua et al. [68], BRCA1 or BRCA2-mutant PDAC were found to harbor increased clusterin-positive cancer-associated fibroblasts (CLU^high^ CAFs) relative to wild-type (WT) tumors. These cells express inflammatory genes such as IL6 and CXCL12 and downregulate other genes like IRF4 and CXCL9 involved in lymphocyte activation and trafficking. BRCA2 mutant models, but not WT counterparts, were able to induce this switch in CAF composition. CAFs incubated with BRCA2-null PDAC cells upregulated the inhibitory checkpoints PDL1, TIGIT, and CD96, adding further support for a distinct immune TME in BRCA1-, BRCA2-, and potentially PALB2-mutated PDAC. Further experiments are needed to validate this hypothesis and explore the potential differential effect of BRCA1, BRCA2, and other HRD phenotypes on the immune TME.

### 4.2. Immunotherapy in BRCA-Mutated Pancreatic Cancer

**Figure 1:**
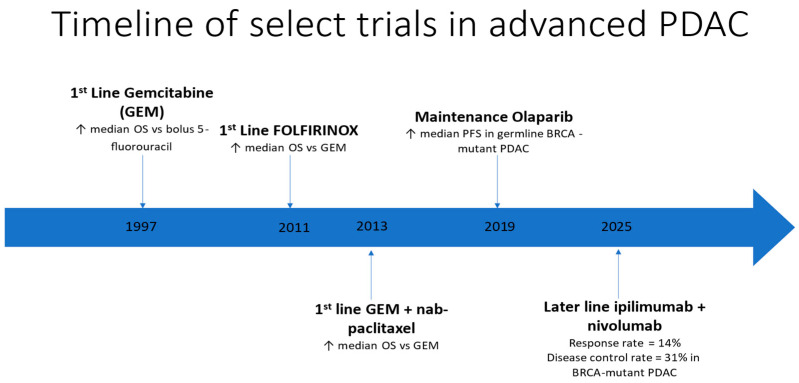
Timeline of select chemotherapeutic and biomarker-directed trials in advanced PDAC.

These experimental findings are also supported by preliminary clinical data. In a retrospective series of 10 patients with PDAC and germline variants in BRCA1, BRCA2, RAD51, or ATM who were treated with combined anti-CTLA4 and anti-PD1 blockade, 30% showed a partial or complete radiographic response and 20% had stable disease for at least 3 months [66]. In the paper by Stossel and colleagues, out of six patients with germline BRCA1 or BRCA2 mutations treated with a checkpoint inhibitor, five progressed but one maintained a complete response for more than 2 years [64]. These early data suggest the potential benefit of an immune-based approach for patients with BRCA-mutated PDAC. The ASCO TAPUR basket trial recently reported results from a cohort of patients with PDAC and BRCA1 or BRCA2 mutations [69]. This prospective single-arm trial enrolled 28 patients and reported a radiographic response rate of 14%, including one patient with a complete response for over 6 years. The response rate in this trial was lower than the report from Terrero et al. [66], possibly because the TAPUR trial included both those with germline and somatic BRCA mutations [69]. Nonetheless, a definite signal of activity was confirmed and met the predefined statistical threshold for further investigation of dual checkpoint blockade in this subgroup. Figure 1 delineates the timeline of select trials in pancreatic cancer.

### 4.3. Future Clinical Approaches

Multiple other approaches to capitalize on the distinct immune TME of HR-deficient PDAC are important to mention. One promising avenue is to combine PARPis with checkpoint inhibition. PARPis can increase CD8^+^ T-cell activity, tumor cell PDL1 expression, and inflammatory cytokine release [70,71]. In a BRCA1-deficient ovarian cancer mouse model, PARPis enhanced the leakage of cytosolic double-stranded DNA, thereby intensifying cGAS-STING pathway activation, and increasing intra-tumoral CD4^+^ and CD8^+^ infiltration at primary and metastatic sites. Similar abilities of PARPis to increase T-cell infiltration and interferon signaling were also reported in a BRCA-null breast cancer model [72,73]. In a recent phase 1b/2 multi-arm non-comparative study, the PARPi niraparib was combined with either nivolumab or ipilimumab in patients with pancreatic cancer with at least stable disease after 4 months of platinum-based chemotherapy; a potential surrogate marker for a BRCAness phenotype. Approximately 15% of patients in each arm harbored germline or somatic mutations in BRCA1, BRCA2, or PALB2. Intriguingly, the median progression-free survival (PFS) was 1.9 months in the nivolumab arm and 8.1 months in the ipilimumab arm. In the subset with BRCA or PALB2 alterations on the niraparib and ipilimumab arm, the median PFS and overall survival were 10.1 and 38 months, respectively [74]. While this study was not powered to compare the two arms, outcomes appeared consistently better with anti-CTLA4 vs. PD1 blockade. Multiple ongoing trials are evaluating the combination of olaparib and a PD1 inhibitor in patients with PDAC and germline or somatic BRCA1, BRCA2, or PALB2 mutations or platinum-sensitive disease (NCT04548752, NCT04493060, and NCT04666740). Larger trials evaluating PARPis with CTLA4 blockade in the maintenance setting are warranted. While only a small subset of patients with advanced PDAC harbor BRCA1, BRCA2, or PALB2 mutations, ongoing efforts are exploring the capacity to induce a BRCAness phenotype in those with wild-type genes. This line of investigation not only has the potential to expand the benefit of PARPis, but also to create a broader population with an immune-permissive TME. Targeting epigenetic pathways via the inhibition of DNA methyltrasferase (DNMT), histone deacetylase, or the enhancer of zeste homolog 2 can impair faithful DNA repair through HR and synergize with PARPis [75]. For example, combining the PARPi talazoparib with low doses of the DNMT inhibitor (DNMTi) azacitidine enhanced the tight and prolonged binding of PARP to chromatin, and resulted in increased double-strand DNA breaks and cell death in triple-negative breast cancer and acute myeloid leukemia cells [76]. Further analyses revealed that azacitidine downregulated several DNA repair-related genes, most prominently in the Fanconi-Anemia pathway [77]. In important follow-up studies, this drug combination induced interferon and inflammasome signaling that was mechanistically linked to the generation of DNA repair defects [50]. It is plausible that this or comparable strategies could also induce an inflamed and immune-sensitive TME and warrants further investigation in pancreatic cancer models. Lastly, with pre-clinical evidence that immune signaling through the cGAS/STING pathway promotes HRD, it is possible that STING agonists could synergize with PARPis in both BRCA- and PALB2-mutated as well as wild-type pancreatic tumors.

## 5. Conclusions

This narrative review highlights current knowledge about the intersection between BRCA mutations, HRD, and the immune microenvironment in PDAC, but many questions remain. Prior work established that biallelic inactivation and core HRD mutations including BRCA and PALB2 are integral for PARPi sensitivity in PDAC [78,79]. Whether these factors or others are also relevant for generating a distinct immune microenvironment is unknown. There is also a need for additional translational studies to better characterize myeloid and T-cell subsets and activity in BRCA- and PALB2-mutated versus wild-type pancreatic cancer. Clinical trials that are exploring novel combination strategies targeting DNA repair in pancreatic cancer should include pre-treatment and on-treatment tissue or liquid-based biopsies to further elucidate the potential relationship between HRD and the immune TME and investigate biomarkers of response and resistance. The TAPUR study provided important prospective data about dual checkpoint blockade in BRCA-mutated PDAC, but larger clinical trials with immune correlates are needed to further characterize the response to immune-based approaches in this subgroup. How a BRCAness phenotype impacts the immune microenvironment and potential sensitivity to currently FDA-approved immunotherapies and novel agents in development will be an important area of research going forward. To summarize, accumulating but still preliminary evidence indicates that BRCA- or PALB2-mutated PDAC harbors a unique and relatively immune-permissive tumor microenvironment. In addition to larger-scale translational and clinical studies, future efforts should also focus on investigating novel combination strategies with agents that induce BRCAness or stimulate the cGAS/STING pathway, with the goal of broadening the population who could benefit from immunotherapeutic approaches in pancreatic cancer.

## Figures and Tables

**Table 1:** Efficacy of platinum-based chemotherapy and PARP inhibitors in BRCA or PALB2-mutated PDAC.

Pt Population	Drug Regimen	Response Rate	Median PFS (Months)	Median OS (Months)
gBRCA/PALB2 1st line	GemCis	74%	10.1	15.5
gBRCA/PALB2 1st line	GemCis + veliparib	65%	9.7	16.4
gBRCA maintenance	Olaparib	20%	7.4	18.9
g/s BRCA/PALB2 maintenance	Rucaparib	42%	13.1	23.5

PFS = progression-free survival; OS = overall survival; g = germline; s = somatic; Gem = gemcitabine; Cis = cisplatin.
